# Measurement properties of patient-reported outcome measures for colorectal cancer: a systematic review

**DOI:** 10.3389/fonc.2026.1789939

**Published:** 2026-04-20

**Authors:** Wen Xu, Yanyan Hong

**Affiliations:** 1 School of Nursing, Nanjing University of Chinese Medicine, Nanjing, China; 2 Department of General Surgery, Nanjing Hospital of Traditional Chinese Medicine, Nanjing, China; 3 Department of Nursing, Nanjing Hospital of Traditional Chinese Medicine, Nanjing, China

**Keywords:** assessment tools, colorectal cancer, evidence-based nursing, measurement properties, patient-reported outcomes, systematic review

## Abstract

**Background:**

Colorectal cancer (CRC) imposes a significant global burden, with approximately 1.93 million new cases diagnosed in 2022. While various patient-reported outcome measures (PROMs) exist to assess the quality of life and symptom burden in this population, their measurement properties vary significantly, particularly between original and translated versions.

**Objective:**

To systematically evaluate the measurement properties and methodological quality of PROMs for patients with colorectal cancer, and to provide evidence-based guidance for instrument selection in clinical practice and research.

**Methods:**

A comprehensive literature search was conducted in Chinese and English databases, including China National Knowledge Infrastructure (CNKI), Wanfang Database, China Science and Technology Journal Database(VIP), China Biomedical Literature Database (CBM), PubMed, CINAHL, Cochrane Library, Web of Science, and Embase, from inception to 31^st^ of January 2026. Two reviewers independently screened studies, extracted data, and assessed methodological quality using the Consensus-based Standards for the selection of health measurement instruments (COSMIN) Risk of Bias checklist. Measurement properties were evaluated according to COSMIN criteria, and the overall quality of evidence was graded using a modified GRADE approach. Instruments were categorized into recommendation levels (A or B) based primarily on evidence for content validity and internal consistency.

**Results:**

Fifteen studies involving 13 colorectal cancer–specific PROMs were included. Overall, the quality of evidence for most instruments was moderate to low, with frequent deficiencies in content validity, structural validity, and responsiveness. Four instruments—Functional Assessment of Cancer Therapy–Colorectal (FACT-C), Memorial Sloan-Kettering Cancer Center Bowel Function Questionnaire (MSKCC-BFQ), Functional Assessment of Cancer Therapy–Colorectal Cancer Symptom Index-9 (FCSI-9), and the Low Anterior Resection Syndrome (LARS) score—demonstrated sufficient content validity and acceptable internal consistency, and were therefore classified as Grade A. The remaining instruments were rated as Grade B due to incomplete psychometric validation or methodological limitations.

**Conclusion:**

Current PROMs for colorectal cancer show variable and generally limited measurement evidence. Original English instruments tend to have more robust psychometric support than Chinese versions and newly developed local scales. FACT-C, MSKCC-BFQ, FCSI-9, and the LARS score can be prioritized for use, while further high-quality validation studies are needed to strengthen the evidence base for other instruments.

**Systematic Review Registration:**

https://www.crd.york.ac.uk/PROSPERO/view/CRD420261278205, identifier CRD420261278205.

## Introduction

According to recent epidemiological data, approximately 1.93 million new cases of colorectal cancer were diagnosed worldwide in 2022, with China ranking second in incidence (about 517,100 new cases) (1). The physical, psychological, and social impairments caused by colorectal cancer and its long-term treatment are substantial and should not be underestimated (2). Patients with colorectal cancer often experience gastrointestinal dysfunction, stoma-related complications, fatigue, anxiety, and depression, all of which can significantly impact quality of life (3).

To accurately capture patients’ subjective experiences, various patient-reported outcome measures (PROMs) have been developed for colorectal cancer, covering symptom burden, physical function, psychological status, and quality of life, thereby supporting individualized and high-quality supportive care (4). However, considerable heterogeneity exists among these instruments with respect to development rigor, psychometric robustness, and cross-cultural applicability. The use of instruments with inadequate quality may lead to inaccurate assessment and inappropriate clinical decision-making (5, 6).

While systematic reviews on PROMs for cancer patients exist, evaluations focusing specifically on colorectal cancer instruments—particularly in the context of cross-cultural adaptation—remain limited. Previous reviews have often focused on English-language instruments or lacked an in-depth evaluation of the instrument development processes and content validity (7). Furthermore, few studies have critically analyzed the discrepancy in psychometric performance between original scales and their translated versions using rigorous methodological standards.

The COSMIN guideline, an internationally recognized standard for evaluating health measurement instruments, provides a comprehensive framework for assessing methodological quality and measurement properties (8). This study aims to bridge the existing knowledge gap by systematically evaluating colorectal cancer–specific PROMs following the COSMIN methodology. By explicitly comparing the performance of original and translated instruments, this review articulates novel insights into the validity of current tools, with the aim of informing clinical practice and guiding future instrument development and adaptation.

## Materials and methods

### Eligibility criteria

#### Inclusion criteria

Studies were included if they met all of the following criteria:

1. Patients diagnosed with colorectal cancer (including colon or rectal cancer).
2. the study involved evaluation of measurement properties (e.g. reliability, validity, responsiveness) of PROMs related to colorectal cancer.
3. instrument development studies, validation studies, or cross-cultural adaptation studies of colorectal cancer PROMs.
4. articles published in Chinese or English.

#### Exclusion criteria

Studies were excluded if they met any of the following criteria:

1. Articles focusing on general health-related quality of life for cancer without a colorectal cancer–specific instrument;
2. Reprints or studies lacking available full-text;
3. Conference abstracts, reviews, commentaries, case reports, or other grey literature.

### Literature search strategy

A systematic search strategy combining subject headings and free-text keywords was implemented for each database. We searched the China National Knowledge Infrastructure (CNKI), Wanfang Database, China Science and Technology Journal Database(VIP), and China Biomedical Literature Database (CBM) for Chinese-language literature, as well as PubMed, CINAHL, Cochrane Library, Web of Science, and Embase for English-language literature. The search timeframe spanned from database inception through 31^st^ of January 2026.The search terms included variations of “colorectal cancer” (e.g., Colorectal Neoplasms, colon cancer, rectal cancer) AND terms for “patient-reported outcomes” (e.g., patient self-report, PROM) AND “instrument” (e.g., scale, questionnaire) AND “psychometric properties”* (e.g., reliability, validity, responsiveness).

The Chinese search query is illustrated with CNKI as an example: (SU= ‘Colorectal cancer’ OR SU= ‘Colorectal tumor’ OR SU= ‘Colorectal cancer’ OR SU= ‘Rectal cancer’) AND (SU= ‘Self-report’ OR SU= ‘Self-evaluation’ OR SU= ‘Patient-reported outcome’) AND (SU= ‘Scale’ OR SU= ‘Questionnaire’ OR SU= ‘Tool’ OR SU= ‘Assessment’ OR SU= ‘Measurement’) AND (SU= ‘Reliability’ OR SU= ‘Validity’ OR SU= ‘Measurement attribute’ OR SU= ‘Internal consistency’ OR SU= ‘Test-retest reliability’).

English search query using PubMed as an example: (((“Colorectal Neoplasms” [MeSH]) OR (“colorectal cancer” [Title/Abstract] OR “colon cancer” [Title/Abstract] OR “rectal cancer” [Title/Abstract] OR “bowel cancer” [Title/Abstract]) AND (“PROM*” [Title/Abstract] OR “patient reported outcome” [Title/Abstract] OR “self-assessment” [Title/Abstract]) AND (“tool” [Title/Abstract] OR “scale*” [Title/Abstract] OR “instrument” [Title/Abstract] OR “questionnaire” [Title/Abstract]) AND (“reliab*” [Title/Abstract] OR “valid*” [Title/Abstract] OR “crosscultural” [Title/Abstract] OR “psychometr*” [Title/Abstract]).

### Study selection and data extraction

All identified records were imported into reference management software, and duplicate entries (identified via NoteExpress) were removed. Two researchers (ZX and HS) independently screened the titles and abstracts of the remaining articles, followed by full-text screening based on the inclusion/exclusion criteria. Any discrepancies in selection were resolved through discussion or by consulting a third reviewer (XW).

For each study that met the inclusion criteria, the following data were extracted: first author, publication year, country, name of the PROM instrument, target patient population, sample size, number of items/dimensions of the instrument, scoring method, time to complete the instrument, and any reported retest (test-retest) interval.

### Quality evaluation

We assessed methodological quality and measurement properties using the COSMIN guidelines. Two researchers independently performed the quality assessments.

### Methodological quality

The methodological quality of each included study was evaluated using the COSMIN Risk of Bias checklist (9). This checklist contains 10 domains (e.g., instrument development, content validity, structural validity, internal consistency, reliability, etc.). Each item is rated on a four-point scale: “Very good,” “Adequate,” “Doubtful,” or “Inadequate.”Following COSMIN guidance, the overall rating for each domain of a study was determined by the lowest rating of any item in that domain (“worst score counts” principle). Thus, if any aspect of a domain was judged inadequate, the entire domain for that study was rated as Inadequate in terms of risk of bias.

### Measurement properties evaluation

We evaluated the measurement properties of each instrument using the COSMIN criteria for good measurement properties (10). Nine measurement properties were considered: content validity, structural validity, internal consistency, cross-cultural validity/measurement invariance, reliability (test-retest or inter-rater reliability), measurement error, criterion validity, hypotheses testing for construct validity, and responsiveness. For each property, the evidence from the studies was rated as “sufficient”, “insufficient”, or “indeterminate” in accordance with COSMIN definitions. If multiple studies assessed the same measurement property for a given instrument and their results were conflicting, we examined the consistency: generally the majority finding was considered, but any substantial inconsistencies were noted and later factored into the evidence grading (see below). In cases of clear disagreement between studies on a property, the finding for that property was marked as inconsistent, which would warrant a downgrade in the quality of evidence for that property.

### Evidence grading and recommendation levels

We applied a modified GRADE approach (11) to rate the overall quality of evidence for each measurement property of each instrument and to derive an overall recommendation. Initially, each measurement property was assumed to have “High” quality evidence. This could be downgraded based on four factors: (1) Risk of bias (methodological quality of the studies, as assessed by COSMIN checklist results), (2) Inconsistency or heterogeneity of results across studies, (3) Indirectness (e.g. evidence not directly generalizable to the target population or context), and (4) other considerations such as imprecision or publication bias (if applicable). After considering these factors, the quality of evidence for each property was categorized as High, Moderate, Low, or Very Low.

Finally, each instrument was assigned a recommendation level (A, B, or C) based on its evidence profile, particularly focusing on its content validity and internal consistency (as these are considered core properties for PROMs):

Level A (Recommended): The instrument has at least sufficient evidence for content validity and for internal consistency. In our criteria, content validity did not require a specific evidence”level” (as long as it was deemed sufficient qualitatively), and internal consistency needed to be supported by at least low-quality evidence (or higher). Instruments meeting these criteria were deemed to have robust support for use in practice.
Level B (Use with caution): The instrument does not fully meet the Level A criteria but still shows acceptable qualities better than Level C. Typically, this means some key properties are supported, but there are remaining concerns or gaps in the evidence (for example, good internal consistency but uncertain construct validity, or vice versa). Such tools can be used in specific contexts with caution, and further validation is advised.
Level C (Not recommended): There is high-quality evidence that at least one critical measurement property is insufficient (e.g., if an instrument has been shown with high confidence to lack content validity or has very poor reliability). Instruments at this level are not recommended for use in clinical practice.

Recommendation levels were determined primarily by evidence for content validity and internal consistency, in accordance with COSMIN guidance, as these are considered core measurement properties for PROMs. Other properties, such as structural validity, reliability, and responsiveness, were incorporated into the grading of evidence certainty and used to inform downgrading decisions and research priorities but were not applied as mandatory criteria for Grade A classification (8).

Detailed justification for the downgrading process, together with a summary mapping of downgrading factors, is provided in the **Supplementary Materials**.

## Results

### Literature search results

**Figure 1:**
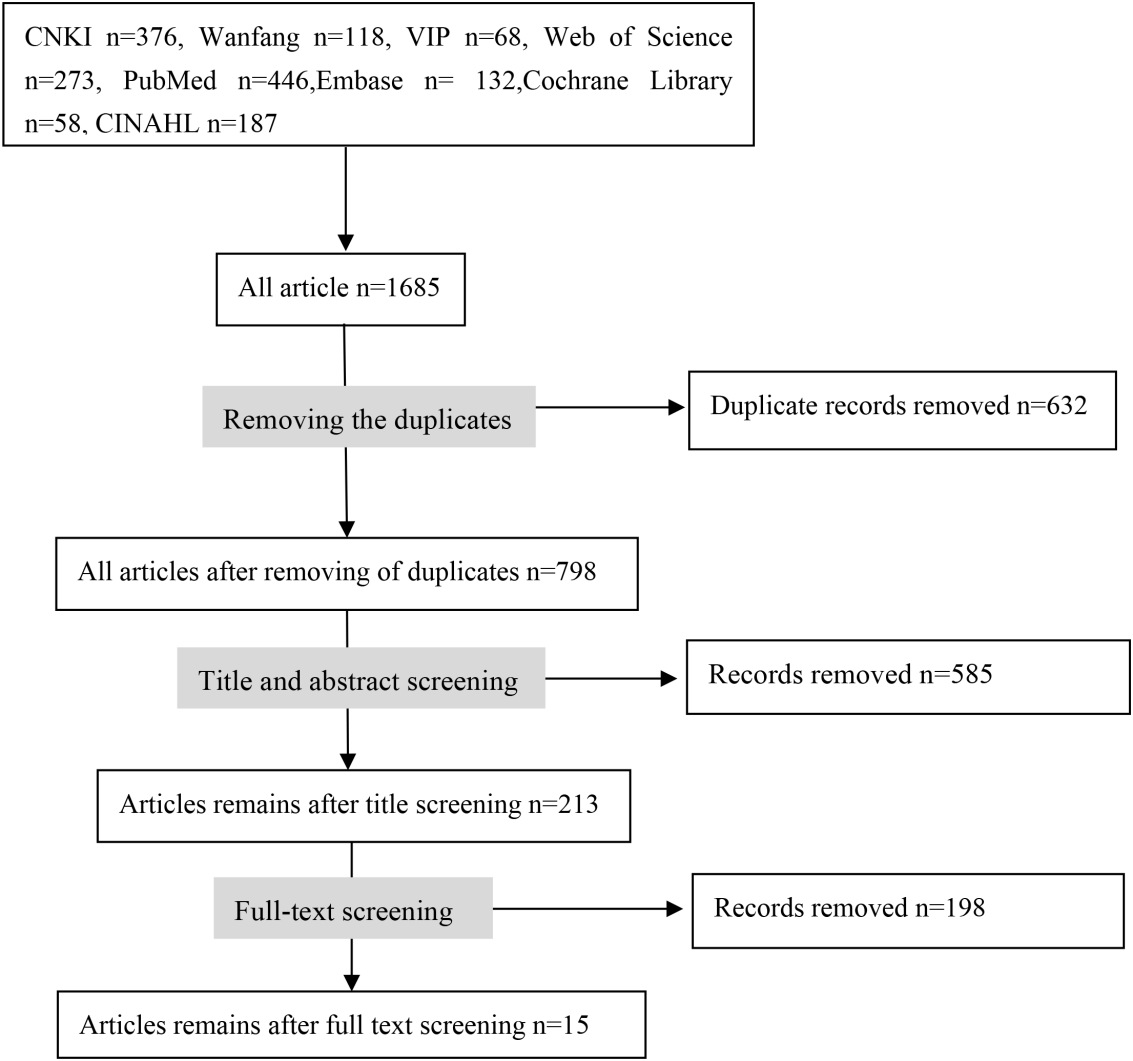
PRISMA flow diagram of the study selection process.

The database search yielded 1,658 records. After removing duplicates and screening titles and abstracts, 228 articles were assessed for full-text eligibility. Ultimately, 15 studies met the inclusion criteria and were included in this systematic review. The study selection process is illustrated in **Figure 1**.

### Characteristics of included studies and instruments

Among the 15 included studies, 8 (12–19) were original instrument development studies and 7 (20–26) were validation or cross-cultural adaptation studies of existing questionnaires (testing translated Chinese versions or culturally adapted versions). In total, 9 distinct colorectal cancer–specific PROMs were identified across these studies: the Functional Assessment of Cancer Therapy-Colorectal (FACT-C) Quality of Life Scale (12, 20, 21), the European Organization for Research and Treatment of Cancer Colorectal Module 29 (EORTC QLQ-CR29) (14, 22, 26), the Quality of Life Instruments for Cancer Patients-Colorectal Cancer (QLICP-CR) (15), and the FACT Colorectal Cancer Symptom Index-9 (FCSI-9) (17). Symptom or function-specific scales: EORTC Quality of Life Questionnaire-Liver Metastases Colorectal 21 (EORTC QLQ-LMC21) (16), Low Anterior Resection Syndrome Score (LARS Score) (24, 25), Memorial Sloan-Kettering Cancer Center Bowel Function Questionnaire (MSKCC-BFQ) (13, 23), Miles’Postoperative Discomfort Scale (18), Permanent Stoma Function Assessment Scale (19).

The key characteristics of each included study (authors, year, country, instrument evaluated, sample size, etc.) are summarized in **Table 1**.

**Table 1:** General characteristics of the included studies (n=15).

Study (First author, Year)	Country	Instrument	Target population	Sample size (n)	Items/Dimensions	Scoring method	Completion time(min)	Retest interval
Ward (1999) (12)	USA	FACT-C	Colorectal cancer patients	134	36/5	Likert 5-point scale (0–4 points)	10	1 week
Temple 2005 (13)	USA	MSKCC-BFQ	Rectal cancer after sphincter-preserving surgery	155	18/3	Likert 5-point scale (1-5points)	10	Not reported
Yost 2005 (20)	USA	FACT-C	Colorectal cancer patients	586	36/5	Likert 5-point scale (0–4 points)	Not reported	Not reported
Gujra 2007 (14)	Multinational	EORTC QLQ-CR29	Colorectal cancer patients	412	29/4	Likert 5-point scale (1–4 points)	Not reported	Not reported
Yang Z 2007 (21)	China	FACT-C (Chinese)	Colorectal cancer patients	110	36/5	Likert 5-point scale (0–4 points)	10~15	24∼48 h
Yang Z 2008 (15)	China	QLICP-CR	Colorectal cancer patients	110	48/5	Likert 5-point scale (1-5points)	Not reported	24∼48 h
Whistance 2009 (22)	Multinational	EORTC QLQ-CR29	Colorectal cancer patients	351	29/4	Likert 5-point scale (1–4 points)	7.5	Not reported
Blazeby 2009 (16)	Britain	EORTC QLQ-LMC21	Patients with colorectal cancer liver metastases	351	21/5	Likert 5-point scale (1–4 points)	5∼10	1~2 weeks
Colwell 2010 (17)	USA	FCSI-9	Colorectal cancer patients	395	9/1	Likert 5-point scale (0–4 points)	<5	2~3weeks
Hou 2014 (23)	China	MSKCC-BFQ (Chinese)	Rectal cancer after sphincter-preserving surgery	102	18/3	Likert 5-point scale (1-5points)	10	2 weeks
Juul 2014 (24)	Multinational	LARS Score	Rectal cancer after sphincter-preserving surgery	1162	5/1	Weight assignment score	Not reported	Not reported
Hou XT 2015 (25)	China	LARS Score (Chinese)	Rectal cancer after sphincter-preserving surgery	186	5/1	Weight assignment score	<5	1–2 weeks
Lin 2017 (26)	China	EORTC QLQ-CR29	Colorectal cancer patients	185	29/5	Likert 5-point scale (1–4 points)	8.5	Not reported
Xiong Y 2020 (18)	China	Miles’Postoperative Discomfort Scale	Postoperative patients of Miles surgery	215	28/5	Likert 5-point scale (0–4 points)	10~15	2 weeks
Yang S 2024 (26)	China	Permanent Stoma Function Assessment Scale	Patients with permanent enterostomy	240	22/4	Likert 5-point scale (1-5points)	10~15 min	2 weeks

### Methodological quality and measurement properties

The evaluation results of methodological quality and measurement attributes of 15 studies are shown in **Table 2**.

**Table 2:** Evaluation results of methodological quality and measurement attributes of included assessment tools (n=15).

Instrument	Dev	CV	SV	IC	CC	Rel	ME	Crit	HT	Resp
FACT-C (12)	A	A/S	A/S	VG/S	D/I	D/Ins	D/Ins	VG/S	VG/S	VG/S
MSKCC-BFQ (13)	VG	VG/S	VG/S	VG/S	NR	VG/S	D/Ins	A/S	VG/S	A/Ins
FACT-C (20)	NR	NR	NR	NR	NR	NR	A/S	NR	VG/S	VG/S
EORTC QLQ-CR29 (14)	VG	VG/S	D/I	D/I	D/I	NR	NR	NR	D/I	NR
FACT-C (Chinese) (21)	A	A/I	A/Ins	VG/S	A/Ins	NR	NR	D/I	A/S	NR
QLICP-CR (15)	A	Inad/S	A/Ins	D/Ins	D/I	VG/S	D/Ins	D/Ins	A/Ins	A/S
EORTC QLQ-CR29 (22)	A	A/S	VG/S	VG/S	VG/S	VG/S	D/Ins	A/S	VG/S	A/S
EORTC QLQ-LMC21 (16)	A	A/Ins	A/S	VG/S	VG/S	VG/S	NR	A/S	D/I	D/Ins
FCSI-9 (17)	A	A/Ins	NR	VG/S	D/I	A/S	D/Ins	A/S	VG/S	VG/S
MSKCC-BFQ (Chinese) (23)	D	D/I	D/Ins	A/Ins	VG/S	A/Ins	NR	D/Ins	A/Ins	D/Ins
LARS score (24)	D	A/Ins	D/Ins	A/Ins	A/S	VG/S	D/Ins	VG/S	A/S	A/S
LARS score (Chinese) (25)	D	D/I	D/Ins	D/I	VG/S	D/I	NR	D/I	D/I	NR
EORTC QLQ-CR29 (Chinese) (26)	NR	D/I	A/S	A/S	A/S	NR	NR	NR	D/Ins	NR
Miles surgery discomfort scale (18)	VG	VG/S	A/S	VG/S	D/Ins	D/Ins	NR	D/Ins	NR/I	A/S
Permanent enterostomy function scale (19)	VG	VG/S	VG/S	VG/S	D/Ins	VG/S	NR	NR	NR	NR

Dev, Development process; CV, Content validity; SV, Structural validity; IC, Internal consistency; CC, Cross-cultural adaptation; Rel, Reliability; ME, Measurement error; Crit, Criterion validity; HT, Hypothesis testing; Resp, Responsiveness; VG, Very good; A, Adequate; D, Doubtful; Inad, Inadequate; S, Sufficient; Ins, Insufficient; I, Indeterminate; NR, Not reported.

### Development process

Four studies (13, 14, 18, 19) explicitly reported the theoretical foundation of scale development, constructing item pools through literature review, patient interviews, and multiple rounds of expert consultations, followed by formal scale formation via pre-testing or item screening. The development process was complete and traceable, thus rated as “Very good.” Five studies (12, 15, 17, 21, 22) explained the scale sources or revision background but provided brief descriptions of item generation, screening, and pre-testing steps, with some merely reporting “item formation based on literature or expert opinions,” resulting in limited process transparency and a rating of “Adequate.” Three studies (23–25) mentioned scale sources or scoring systems but failed to determine whether a systematic development process was actually conducted, with insufficient methodological information, leading to a rating of “Doubtful.”

### Content validity

Methodological quality assessment: Six studies (13, 14, 17–19, 24) explicitly described expert selection criteria (typically 5–15 experts), expert composition (covering clinical medicine, nursing, and scale development), and consultation processes (e.g., Delphi or structured expert review), and were evaluated using item relevance scores or content validity indices, with methodological standards rated as “Very good”; Four studies (12, 16, 21, 22) reported expert consultations or item discussion processes but did not specify expert qualifications or report item-level relevance evaluation methods, rated as “good”; Three studies (23, 25, 26) mentioned item revisions or discussions but did not clearly describe the evaluation processes for item relevance, comprehensiveness, or comprehensibility, rated as “Doubtful”.

Measurement attribute evaluation: Expert or target population feedback from 6 studies (12–14, 18, 19, 22) explicitly supported the relevance and comprehensiveness of the items, rated as “sufficient”; 4 studies (16, 17, 21, 24) conducted content validity evaluations, but some items showed low relevance scores or lacked quantitative support, rated as “insufficient”; the remaining studies were rated as “Indeterminate”.

### Structural validity

Methodological quality assessment: Five studies (13, 16, 18, 19, 22) systematically examined the scale structure using exploratory factor analysis (principal component analysis or principal axis factor method, Kaiser–Meyer–Olkin value>0.70, Bartlett’s test P<0.05) or confirmatory factor analysis (reporting fit indices such as Comparative Fit Index ≥0.90), rated as “Very good”; four studies (12, 15, 21, 26) supported the rationality of the scale structure through item–dimension correlation coefficients or indirect evidence of known structure, but did not fully report the factor analysis process, rated as “good”; four studies (14, 23–25) mentioned the scale dimension settings but did not report the method of standard structural validity testing, rated as “Doubtful”.

Measurement attribute evaluation: Factor analysis results from 6 studies (12, 13, 16, 18, 19, 22) supported the established dimensional structure (principal factor loadings ≥0.40), rated as “sufficient”; factor cross-loadings or dimensional instability were observed in 5 studies (15, 21, 23, 24, 26), rated as “insufficient”. The remaining studies were rated as “Indeterminate”.

### Internal consistency

Methodological quality assessment: Seven studies (12, 13, 15–19, 21, 22) reported Cronbach’s α coefficients, with most dimensions showing α values within the 0.70–0.90 range. The statistical methods were standardized and rated as “Very good”. Three studies (23, 24, 26) reported internal consistency results, but some dimensions had α values close to or slightly below 0.70, or the sample sizes were limited, and were rated as “Adequate”. Three studies (14, 15, 25) mentioned scale reliability but did not clearly distinguish between internal consistency analysis methods and were rated as “Doubtful”.

Measurement attribute evaluation: The Cronbach’s α values of 8 studies (12, 13, 16–19, 21–23) met the recommended threshold (≥0.70) and were rated as “sufficient”; 4 studies (15, 23, 24, 26) had at least one dimension with an α value <0.70 and were rated as “insufficient”; the remaining studies were rated as “Indeterminate”.

### Cross-cultural validity

Methodological quality assessment: Four studies (16, 22, 23, 25) explicitly described forward translation, back-translation, expert committee review, and pilot testing procedures, meeting cross-cultural adaptation criteria and were rated as “Very good”; three studies (21, 24, 26) mentioned translation or cultural adaptation but had incomplete procedures and were rated as “Adequate”; five studies (12, 14, 15, 17, 19) reported insufficient information on cultural adaptation and were rated as “Doubtful”.

Measurement attribute evaluation: The translation process, pre-test results, and subsequent measurement performance in five studies (16, 22–25) consistently supported the semantic equivalence and cultural appropriateness of the scale in the target culture, rated as “sufficient”; three studies (19, 21, 26) showed partial cultural inappropriateness or dimensional structure changes after translation, rated as “insufficient”; the remaining studies were rated as “Indeterminate”.

### Reliability

Methodological quality assessment: Five studies (13, 15, 16, 19, 22) reported retest intervals (mostly 1–2 weeks) and used appropriate indicators such as intraclass correlation coefficients to evaluate scale stability, rated as “Very good”; three studies (17, 23, 24) reported good reliability indicators but did not specify retest intervals or sample sizes, rated as “Adequate”; four studies (12, 14, 18, 25) mentioned scale stability but did not report standardized retest analyses, rated as “Doubtful”.

Measurement attribute evaluation: In 6 studies (13, 15–17, 19, 22), the intraclass correlation coefficient or correlation coefficient ≥0.70 was rated as “sufficient”; in 3 studies (18, 23, 24), the stability coefficient did not meet the recommended threshold and was rated as “insufficient”; the remaining studies were rated as “Indeterminate”.

### Measurement error

Methodological quality assessment: One study (20) used the distribution method to estimate standard errors and minimum significant differences, rated as “Adequate”; four studies (12, 13, 17, 24) mentioned change judgment or measurement precision but did not report standard errors or minimum detectable changes, rated as “Doubtful”.

Measurement attribute evaluation: The results of one study (20) supported adequate measurement error control, rated as “sufficient”; three studies (12, 13, 24) reported variation indicators but failed to meet the minimum detectable variation requirement, rated as “insufficient”; the remaining studies were rated as “Indeterminate”.

### Criterion validity

Methodological quality assessment: Two studies (12, 24) explicitly used external scales or clinical outcomes as calibration criteria, reporting correlation coefficients or discriminant ability, and were rated as “Very good”; four studies (13, 16, 17, 22) indirectly supported calibration criterion validity through correlation analysis with related scales, and were rated as “good”; four studies (15, 21, 23, 25) mentioned comparisons with other indicators but did not specify calibration criteria, and were rated as “Doubtful”.

Measurement attribute evaluation: The correlation coefficients of 5 studies (12, 13, 16, 17, 22) reached the expected direction and strength, rated as “sufficient”; the remaining studies were rated as “Indeterminate”.

### Hypothesis testing

Methodological quality assessment: Five studies (12, 13, 16, 17, 22) explicitly hypothesized and validated through known-group comparisons or correlation analyses, rated as “Very good”; four studies (15, 21, 23, 24) indirectly supported the hypotheses, rated as “good”; three studies (14, 25, 26) mentioned group differences but failed to establish a complete hypothesis testing framework, rated as “Doubtful”.

Measurement attribute evaluation: The results of 7 studies (12, 13, 16, 17, 21, 22, 24) were consistent with the prespecified hypotheses and were rated as “sufficient”; the correlation coefficients of 4 studies (15, 18, 23, 26) were low or unstable and were rated as “insufficient”; the remaining studies were rated as “Indeterminate”.

### Responsiveness

Methodological quality assessment: Four studies (12, 17, 20, 22) evaluated responsiveness using pre-and post-treatment comparisons, standardized mean response, or anchoring methods, rated as “Very good”; three studies (13, 15, 24) indirectly reflected responsiveness through pre-and post-treatment differences, rated as “Adequate”; three studies (16, 18, 23) mentioned trends but did not report standardized response metrics, rated as “Doubtful”.

Measurement attribute evaluation: The responsiveness indices of 6 studies (12, 15, 17, 20, 22, 24) reached clinically interpretable levels and were rated as “sufficient”; 5 studies (13, 16, 18, 19, 23) were insensitive to clinical changes and were rated as “insufficient”; the remaining studies were rated as “Indeterminate”.

### Evidence quality and recommendations

Based on the synthesis of evidence from all studies, we graded the overall quality of evidence for each instrument’s key properties and assigned an overall recommendation level (A or B) for each PROM (see **Table 3** for a summary).

**Table 3:** Summary of recommendation levels of colorectal cancer–specific assessment tools (n=13).

Instrument	CV	SV	IC	CC	Rel	ME	Crit	HT	Resp	Rec
FACT-C (12, 20)	A/Moderate	A/Low	VG/Moderate	NR	NR	NR	I/Low	VG/Moderate	VG/Moderate	A
MSKCC-BFQ (13)	VG/Moderate	VG/Moderate	VG/Moderate	NR	VG/Moderate	NR	NR	A/Moderate	I/Low	A
EORTC QLQ-CR29 (14, 22)	VG/Moderate	I/Low	I/Low	NR	NR	NR	NR	I/Low	NR	B
FACT-C (Chinese) (21)	A/Low	I/Low	VG/Moderate	A/Low	NR	NR	NR	A/Low	NR	B
QLICP-CR (15)	Ins/Low	I/Low	I/Low	NR	NR	NR	NR	I/Low	NR	B
EORTC QLQ-LMC21 (16)	A/Low	I/Low	A/Low	NR	NR	NR	NR	I/Low	NR	B
FCSI-9 (17)	A/Moderate	NR	VG/Moderate	NR	A/Low	I/Low	A/Low	VG/Moderate	VG/Moderate	A
MSKCC-BFQ (Chinese) (23)	D/Low	I/Low	Ins/Low	VG/Low	A/Low	NR	NR	A/Low	I/Low	B
LARS score (24)	A/Moderate	NR	NR	NR	VG/Moderate	NR	I/Low	VG/Moderate	I/Low	A
LARS score (Chinese) (25)	A/Low	NR	NR	VG/Low	A/Low	NR	I/Low	A/Low	I/Low	B
EORTC QLQ-CR29 (Chinese) (26)	D/Low	I/Low	Ins/Low	A/Low	NR	NR	NR	I/Low	NR	B
Discomfort Scale after Miles Surgery (18)	VG/Low	I/Low	A/Low	NR	I/Low	NR	NR	NR	I/Low	B
Functional Assessment Scale for Permanent Enterostomy (19)	VG/Low	I/Low	A/Low	NR	VG/Low	NR	NR	NR	NR	B

CV, Content validity; SV, Structural validity; IC, Internal consistency; CC, Cross-cultural adaptation; Rel, Reliability/stability; ME, Measurement error; Crit, Criterion validity; HT, Hypothesis testing; Resp, Responsiveness; Rec, Recommendation level; VG, Very good; A, Adequate; D, Doubtful; I, Indeterminate; Ins, Insufficient; NR, Not reported.

### Evidence grading

The certainty of evidence for the measurement properties of patient-reported outcome measures was assessed using a modified GRADE approach in accordance with COSMIN recommendations (8, 11). Evidence downgrading was applied consistently across instruments based on predefined methodological domains rather than instrument-specific performance comparisons.

Downgrading was primarily driven by recurrent methodological limitations observed in the primary validation studies, including insufficient patient involvement in content validation, limited or indirect evidence for structural validity, small sample sizes leading to imprecision, and incomplete reporting of key psychometric indices such as SEM, SDC, or ICC. In several cases, acceptable numerical results (e.g., Cronbach’s α values) were interpreted with caution when underlying assumptions—such as confirmed unidimensionality—were not adequately supported.

Importantly, evidence downgrading reflects limitations in the strength and completeness of the available evidence rather than deficiencies in the clinical relevance or practical applicability of the instruments themselves. Final recommendation levels were determined by synthesizing evidence across measurement properties and remain unchanged despite variability in evidence certainty.

A transparent overview of the downgrading framework and its methodological basis is provided in the Supplementary Appendix, including a summary mapping of downgrading factors across measurement properties (**Supplementary Table 1**).

## Discussion

### Content validity: gaps in evidence

Our evaluation revealed that content validity is the most frequently under-addressed measurement property among colorectal cancer PROMs (27). Many of the reviewed instruments (12, 14, 17, 22, 24, 25) were found to have “Doubtful” ratings in content validity due to insufficient documentation or methodology in the development phase. For instance, while most tools mentioned that expert input was sought during item generation or revision, the details were often lacking – e.g. the criteria for selecting experts, the procedures for the expert panel or interviews, and how item relevance was quantitatively assessed were seldom fully reported. This lack of transparency and rigor resulted in lower confidence in whether these instruments truly capture the full breadth and depth of relevant patient-reported outcomes. Only a few tools (13, 17) explicitly described robust content validation processes, such as integrating literature reviews, patient interviews, and multiple rounds of expert consultation to ensure all important concepts were covered. These instruments correspondingly received stronger content validity ratings.

In contrast, some scales developed locally in China (15, 18, 19) relied heavily on investigators’ personal clinical experience or limited advisory discussions when generating items. Such approaches, without systematic qualitative methods, may miss important patient concerns or include irrelevant items, leading to content that may not resonate fully with the patient experience. Our findings underscore that content validity is both critical and currently a weak link in many PROMs. Improving the upfront development process by thoroughly involving patients and experts and clearly documenting these steps is essential for future instrument development.

### Cultural and systemic biases in instrument design

A critical finding of this review is the discrepancy in structural validity between original English instruments and their translated or locally developed Chinese counterparts. While original instruments generally underwent extensive factor analyses confirming their dimensional structure (12, 13, 16, 22), Chinese versions frequently exhibited “Indeterminate” or “Insufficient” structural validity (15, 19, 21, 23, 26). This disparity suggests potential cultural or systemic biases in the adaptation process. The deductive approach often used in translation—where items are translated literally without sufficient cultural adaptation—may result in items that do not conceptually align with the Chinese patient experience (21, 23, 26). Furthermore, locally developed scales often relied on clinical experience rather than the rigorous, inductive qualitative research (e.g., patient focus groups) required by COSMIN to generate culturally relevant items (15, 18, 19). This lack of deep cultural adaptation likely contributes to the structural instability observed when these tools are subjected to factor analysis.

### Structural validity vs. internal consistency: an unbalanced emphasis

We observed an interesting contrast between structural validity and internal consistency evidence among the instruments. Structural validity (the degree to which the items of an instrument reflect the dimensionality of the construct, usually assessed via factor analysis) showed marked disparities between instruments. Several original English instruments (12, 22), underwent extensive exploratory or confirmatory factor analyses with generally favorable outcomes, providing relatively solid evidence that their items indeed group into the intended domains. On the other hand, many translated Chinese versions and new instruments developed in Chinese (19, 21, 26) had either not performed rigorous factor analyses or did not fully report the results (e.g., factor loadings, model fit indices). Consequently, the structural validity evidence for those tools was limited. In some cases, the factor structure of the Chinese versions deviated from the original (possibly due to cultural differences in item interpretation or translation issues), but sample sizes were often too small to draw firm conclusions, leading to an “Indeterminate” rating for structural validity in a number of cases.

In contrast, internal consistency (often measured by Cronbach’s alpha) was reported in nearly all studies and generally received adequate attention. Multiple scales (13, 17, 21) reported Cronbach’s α coefficients that met or approached the commonly recommended threshold of 0.70–0.80 for group comparisons. In fact, for many instruments, all subscales had alpha values indicating acceptable to excellent internal consistency, reflecting a high degree of inter-relatedness among items within the same domain. However, while a high Cronbach’s alpha is desirable, it alone does not guarantee a well-constructed instrument. Internal consistency simply indicates that items correlate with each other, but it does not confirm that they collectively measure the correct underlying construct (28). Some instruments, particularly those with high alpha but uncertain factor structure, illustrate this point. For example, a couple of scales (21, 26) showed good internal consistency yet only adequate or even indeterminate structural validity evidence. In such cases, COSMIN and measurement theory caution that strong internal consistency cannot compensate for poor structural validity; if the items are not confirmed to belong together (i.e., measure the same construct), a high alpha might be artificially inflated (for instance, by redundant items) and may not reflect true unidimensionality. This is one reason why despite decent reliability statistics, some instruments (23, 26) in our review remained categorized with an overall “Indeterminate” or only Grade B recommendation – their underlying construct validity was in question. In summary, developers of PROMs should strive for a balance: ensuring a sound theoretical and factor-analytic basis for the scale (structural validity) and high internal consistency. Over-reliance on Cronbach’s alpha without confirming factor structure can be misleading.

### Reliability and responsiveness: limited reporting

Two other measurement properties that are crucial for clinical utility–test–retest reliability and responsiveness–were under-reported in many studies. Test–retest reliability assesses whether an instrument can produce consistent scores over time in stable patients (29). In our review, only a subset of studies (13, 19, 24) explicitly reported a test–retest analysis or intraclass correlation coefficients (ICCs). Those that did often had adequate methods (e.g., an appropriate time interval where no change in patient condition is expected). For example, the MSKCC-BFQ and LARS score had test–retest ICCs in the “Very good” range (often >0.85) when assessed. However, many studies either did not include a retest or had methodological flaws such as too short or too long an interval, or they simply stated “reliability” by Cronbach’s alpha (internal consistency) instead of an actual stability measure. As a result, the reliability evidence for several instruments was marked as “Doubtful” or “Indeterminate,” and the quality of evidence was downgraded accordingly. This gap indicates that more prospective studies should incorporate repeat administrations of the PROM in a stable patient subset to confirm that the instrument yields reproducible results.

Responsiveness, defined as the instrument’s ability to detect clinically important changes over time, was the least reported property across the board (29). Only two instruments (12, 17) provided clear evidence of responsiveness in our included studies. For instance, FACT-C was shown in one study to change in expected directions following interventions, and minimal important difference values had been estimated for it. The FCSI-9, being a symptom index, also demonstrated score changes corresponding to patient-reported symptom improvement or deterioration. Most other instruments either did not include any longitudinal analysis or the studies were cross-sectional only. In some cases, responsiveness might not have been applicable if the study design did not involve an intervention or follow-up. Nonetheless, for an instrument to be useful in evaluating treatment outcomes or disease progression, responsiveness is key. The lack of responsiveness testing significantly limits the confidence in using many of these PROMs for longitudinal monitoring in clinical trials or practice. For example, if a questionnaire has good reliability and validity but we do not know if it can pick up meaningful changes after surgery or chemotherapy, its utility for outcome assessment is questionable. Our findings suggest an important direction for future research: conducting longitudinal studies to assess how sensitive each PROM is to changes in patient status, such as post-treatment recovery or disease recurrence.

### Quality of evidence grading and recommendations

Using the modified GRADE approach provided a structured way to aggregate findings and make recommendations for each instrument. Grade A instruments (FACT-C (12, 20), MSKCC-BFQ (13), FCSI-9 (17), LARS score (24)) generally had consistent evidence of adequate content validity and at least one other key attribute with no major concerns. These instruments can be considered as having “high evidence support” at present. In practical terms, clinicians or researchers selecting a PROM for colorectal cancer patients can have greater confidence in these tools for providing reliable and valid patient-reported data. For instance, FACT-C and MSKCC-BFQ have been used internationally and have accumulated supportive evidence, which our review confirms. It is worth noting that even Grade A tools are not perfect – they simply have fewer and less severe limitations in current evidence. Ongoing evaluation and refinement can further strengthen these instruments (for example, by updating content or adding modules for new treatments).

Grade B instruments, which comprised the majority of the tools in our review, highlight the reality that “medium” quality evidence is more common than high quality in this field. Many of these tools have one or two strong points but also at least one notable weakness. For example, the EORTC QLQ-CR29 (14, 22) is well-validated in its original form and covers important quality of life aspects, but our review noted issues in its content validity evaluation and some inconsistent results in different cultural contexts, which prevented it from being Grade A despite its popularity. Similarly, QLICP-CR (15) is a comprehensive instrument designed for Chinese patients, but it lacks external validation and has methodological shortcomings in its development that lower our confidence in it. For all Grade B instruments, we recommend caution: they should be used in scenarios where they have shown some effectiveness, and users should be mindful of their limitations.

Importantly, a lower recommendation grade does not equate to dismissing the instrument entirely. A Grade B tool may still be the best available option for a particular subdomain or setting (especially if no Grade A tool exists for that niche). The grading reflects the strength of current evidence, not necessarily the intrinsic worth of the instrument. Some Grade B instruments could be upgraded in the future if new, high-quality studies address the gaps (for example, a thorough content validity study or a large multicenter validation could elevate an instrument’s status). On the flip side, practitioners should avoid over-relying on Grade B tools for critical decisions without corroborating information, since the evidence backing those tools is incomplete. To strengthen future evidence-based PROM selection, COSMIN scoring and recent COSMIN-based syntheses should be considered when selecting instruments and interpreting measurement-property evidence (30–32).

### Limitations

This review was limited to studies published in Chinese or English, which may introduce some publication bias and language bias in our findings. It is possible that relevant validation studies in other languages or unpublished data were not captured. Despite this, our comprehensive search and rigorous evaluation framework provide a valuable overview of the current state of colorectal cancer PROMs and highlight areas in need of improvement.

## Conclusion

In this systematic review of colorectal cancer patient-reported outcome measures, we found that existing instruments still exhibit deficiencies in several key measurement properties, notably in content validity, structural validity, and responsiveness. Overall, the quality of evidence supporting these PROMs is predominantly at a moderate to low level. The original English versions of certain core instruments (such as FACT-C and others) tend to have more robust psychometric evidence, whereas most Chinese versions and newly developed local scales remain in need of further validation and refinement. Among the tools evaluated, FACT-C, MSKCC-BFQ, FCSI-9, and the LARS score emerged as the instruments with relatively superior comprehensive measurement performance, representing the highest level of current evidence support for use in colorectal cancer patients. These instruments can be prioritized as preferred options for assessing patient-reported outcomes in both clinical practice and research settings.

Looking forward, there is a clear need for ongoing efforts to develop and improve PROMs for colorectal cancer. Future research should adhere closely to COSMIN guidelines and best practices, ensuring thorough content validation (with patient involvement), robust psychometric testing (including factor analysis, reliability, and responsiveness assessments), and cross-cultural validation for translated versions. By doing so, we can achieve higher-quality PROMs that are both scientifically sound and culturally relevant to the target population. High-quality, well-validated instruments will provide more reliable patient-centered data to inform clinical decisions, evaluate interventions, and ultimately improve supportive care for colorectal cancer patients.

## Data Availability

The original contributions presented in the study are included in the article/**Supplementary Material**. Further inquiries can be directed to the corresponding author.
