# Supplementary material for: Measurement properties of patient-reported outcome measures for colorectal cancer: a systematic review
Source: Front Oncol. 2026 Apr 20;16:1789939. doi: 10.3389/fonc.2026.1789939 (PMC13137305; doi:10.3389/fonc.2026.1789939)
Supplement: Supplementary file 1 [file DataSheet1.pdf]

To enhance the transparency and reproducibility of the evidence quality assessments, this Supplementary Appendix provides structured and explicit justifications for all downgrading decisions applied in the modified GRADE process.

For each patient-reported outcome measure and each evaluated measurement property, downgrading decisions were made in accordance with the COSMIN methodology and were based on one or more of the following predefined factors:

(1) Risk of bias, as determined by the COSMIN Risk of Bias checklist (e.g., inadequate structural validation procedures, lack of patient involvement in content validation, or suboptimal statistical methods);

(2) Inconsistency, reflected by conflicting findings across studies or instability of measurement structure;

(3) Imprecision, primarily due to limited sample sizes or insufficient statistical power; and

(4) Incomplete reporting, where key indicators (e.g., SEM, SDC, ICC) required for robust interpretation were not available.

Table S1. Summary of Evidence Downgrading Factors in the Modified GRADE Assessment

Measurement property	Primary downgrading factor (GRADE)	COSMIN-related methodological issue	Source of limitation
Content validity	Risk of bias	Limited or insufficiently reported patient involvement in qualitative evaluation of item relevance, comprehensiveness, and comprehensibility	Incomplete reporting of cognitive interviews or patient debriefing in primary studies
Structural validity	Risk of bias / Imprecision	Absence of confirmatory factor analysis or reliance on exploratory or indirect methods; in some cases, limited sample size for robust structural confirmation	Validation studies did not consistently perform CFA or were underpowered
Internal consistency	Indirectness	Interpretation dependent on insufficiently established structural validity and unverified unidimensionality	Structural validity evidence was limited or indirect
Reliability	Risk of bias	Use of correlation coefficients instead of	Statistical methods reported in primary

		intraclass correlation coefficients, limiting assessment of systematic measurement error	studies
Measurement error	Incomplete reporting	Key absolute measurement error indices (e.g., SEM, SDC) not reported	Primary studies did not provide measurement error parameters
Cross-cultural validity	Risk of bias / Incomplete reporting	Translation or cultural adaptation procedures insufficiently detailed or lacking multi-group validation	Limited reporting of cross-cultural adaptation processes
Hypothesis testing	Imprecision	Limited sample size or unstable correlation estimates in known-groups or convergent validity testing	Sample size constraints or incomplete reporting
Responsiveness	Imprecision / Incomplete reporting	Absence of standardized responsiveness indices or limited longitudinal follow-up	Longitudinal analyses not consistently conducted or reported

Each downgrading rationale reported in the Supplementary Table corresponds directly to one or more of these criteria and is intended to provide a clear audit trail linking primary study characteristics to evidence quality judgments. Importantly, downgrading reflects limitations in the strength and completeness of the available evidence, rather than deficiencies in the clinical usefulness or applicability of the instruments.
